# *Banhahubak*-Tang Tablet, a Standardized Medicine Attenuates Allergic Asthma via Inhibition of Janus Kinase 1 (JAK1)/ Signal Transducer and Activator of Transcription 6 (STAT6) Signal Pathway

**DOI:** 10.3390/molecules25092206

**Published:** 2020-05-08

**Authors:** Yeon Kyung Nam, Seong Chul Jin, Mi Hye Kim, La Yoon Choi, Yong-Bok Lee, Woong Mo Yang

**Affiliations:** 1 Department of Convergence Korean Medical Science, College of Korean Medicine, Kyung Hee University, Seoul 02447, Korea; nyk7705@khu.ac.kr (Y.K.N.); misterjin23@khu.ac.kr (S.C.J.); kimmihye526@khu.ac.kr (M.H.K.); lydanas@khu.ac.kr (L.Y.C.); 2 College of Pharmacy, Chonnam National University, Gwangju 61186, Korea; leeyb@chonnam.ac.kr

**Keywords:** allergic asthma, BHT, PM_10_, airway inflammation, pro-inflammatory cytokine, Th2-related cytokine

## Abstract

Exposure to particulate matter (PM) has been known to be one of the risk factors to cause allergic asthma, leading to development of respiratory disease. *Banhahubak*-tang tablet (BHT), a standardized Korean Medicine, is prescribed for neurasthenia, laryngopharyngitis and asthma. In this study, we investigated therapeutic effects of BHT on airway inflammation in ovalbumin (OVA) and PM smaller than 10 μm (PM_10_)-induced allergic asthma mice. To establish allergic asthma with airway hyper-responsiveness by PM_10_, BALB/c mice were sensitized and challenged with OVA and PM_10_, and orally administered BHT. Histological staining was performed to assess airway remodeling. Serum and bronchoalveolar lavage fluid (BALF) was collected for measuring immunoglobulin levels and counting inflammatory cells, respectively. Expression levels of Janus kinase 1 (JAK1)/signal transducer and activator of transcription 6 (STAT6), pro-inflammatory cytokines and type 2 T-helper (Th2)-related cytokines were analyzed in vivo and in vitro models. Histopathological analysis demonstrated that BHT suppressed inflammatory cell infiltration, mucus hypersecretion and collagen deposition in the airway. BHT administration effectively decreased number of inflammatory cells in BALF. BHT reduced total serum Immunoglobulin E (IgE) and Immunoglobulin G (IgG) levels. In addition, BHT significantly inhibited the phosphorylation of JAK1 and STAT6 expressions. Release of pro-inflammatory cytokines and Th2-related cytokines were down-regulated by BHT. In conclusion, BHT mitigated airway inflammation by down-regulating pro-inflammatory and Th2-related cytokines via JAK1/STAT6 signaling. BHT might be a promising herbal medicine for preventing airway inflammation. Moreover, an intervention study among humans is needed to further evaluate the possible beneficial effects of BHT in allergic asthma.

## 1. Introduction

Allergic asthma is classified as a common bronchial disorder, accompanying symptoms such as recurrent coughing, wheezing and dyspnea [1]. According to the medical consultation statistics, morbidity and prevalence rate of allergic asthma is rapidly increased [2]. Allergens, respiratory infection and air pollution are risk factors that predispose people to develop allergic asthma [3]. Consecutive exposure to allergen leads to bronchoconstriction and airflow narrowing, which are clinically characterized by airway hyperactivity, goblet cell hyperplasia and airway remodeling [4,5]. Among many risk factors, fine dust that is particulate matter (PM), which is smaller than 10 μm (PM_10_) can stimulate various health problems including neurodegenerative, cardiovascular and respiratory diseases. PM_10_ penetrates lung in depth and accrues to the alveoli leading to lung dysfunction [3,6].

PM_10_ is reported to induce secretion of pro-inflammatory cytokines such as tumor necrosis factor-α (TNF-α), interleukin (IL)-1β, IL-6, IL-8 and IL-17A from airway epithelial cells following activation of macrophage and T-cells [7]. In addition, PM_10_ has an ability to impede differentiation of type 2 T-helper (Th2) cell, which releases Th2-related cytokines, IL-4, IL-5 and IL-13 [8]. To establish an allergic asthma model in our study, sensitization and ovalbumin (OVA) challenge with adjuvant were required to express properties of the in vivo allergic asthma model [9]. Considering that PM_10_ is able to penetrate lung bronchiole, PM_10_ was instilled into the nasal for the challenge process.

Treatment for allergic asthma is aimed to prevent various asthmatic symptoms; two types include: quick-relievers and long-term controllers [10]. Bronchodilators, including albuterol, terbutaline and ipratropium, are non-steroidal quick-relievers, which rapidly expand the contracted bronchial muscle [11]. Although the bronchodilator is known to be helpful for steady breathing in the short-term, it is difficult to settle fundamental issues that set off the asthmatic crisis [12]. Long-term controllers, such as inhaled corticosteroids (ICS), have been used, for several years, as effective agents that mitigate airway inflammation [13]. In addition, using ICS with long acting beta 2 agonists (LABA) is recommended for patients with severe asthma [14]. However, those drugs could cause adverse effects, such as tachycardia, skeletal muscle tremor and hypokalemia [15]. Therefore, interest has risen in alternative treatments for management of allergic asthma. In particular, herbal medicines with minimal side effects and improved remedial effects, are used in the strategy of asthmatic symptoms.

*Banhahubak*-tang tablet (BHT), a standardized Korean Medicine, is formulated from six herbs containing *Pinellia ternata* (Thunb.) Makino (Araceae), *Magnolia officinalis* Rehder et E. H. Wilson (Magnoliaceae), *Poria cocos* F.A.Wolf (Polyporaceae), *Perilla frutescens* Britton var. crispa Decne (Labiatae), *Zingiber officinale* Roscoe (Zingiberaceae) and *Ziziphus jujube* Mill. (Rhamnaceae). Although BHT is prescribed for neurasthenia, dyspepsia and bronchial asthma, there is little experimental research regarding the effect of BHT on PM_10_-induced respiratory diseases [16,17]. Therefore, we investigated anti-asthmatic and inhibitory effects of BHT by establishing OVA + PM_10_-induced allergic asthma mice. Furthermore, we evaluated whether BHT ameliorates the expression levels of pro-inflammatory cytokines and Th2-related cytokines through Janus kinase 1 (JAK1)/signal transducer and activator of transcription 6 (STAT6) pathway in in vivo and in vitro.

## 2. Results

### 2.1. Effect of BHT on Airway Remodeling in OVA and PM_10_-Induced Mice

**Figure 1:**
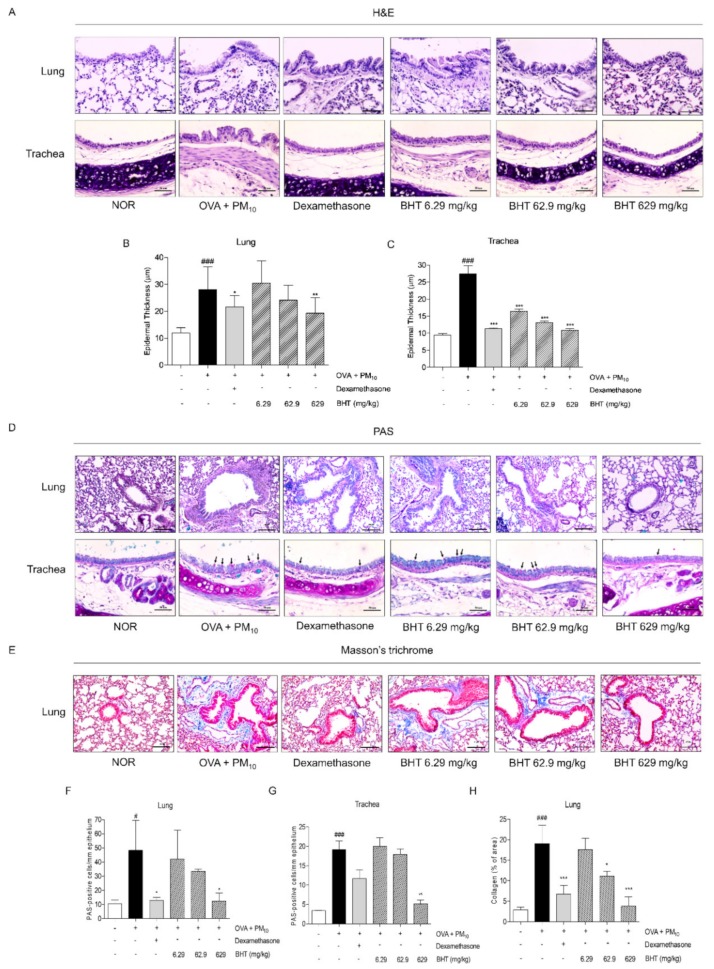
Histopathological analysis for observing airway remodeling. (**A**–**C**) Representative images of hematoxylin and eosin staining for measuring epithelial thickness in lung and trachea tissues (Magnification 400×, scale bar 100 μm). (**D**,**F**,**G**) Periodic Acid–Schiff staining for counting goblet cells in lung and trachea tissues (Magnification 400×, scale bar 50 μm). Black arrows: goblet cell (**E**,**H**) Masson′s trichrome staining for measuring amount of collagen deposition in lung tissues (Magnification 100x, scale bar 200 μm). Quantitative data was analyzed by image J program. Statistical results are presented as the mean ± SEM. ^#^ *p* < 0.05 and ^###^ *p* < 0.001 compared to normal saline (NOR) group; * *p* < 0.05, ** *p* < 0.01 and *** *p* < 0.001 compared to the ovalbumin (OVA) + particulate matter (PM) group.

After sacrifice, lung and trachea tissues were collected. Hematoxylin and eosin (H&E), Periodic Acid-Schiff (PAS) and Masson′s trichrome staining were performed to evaluate airway remodeling in lung and trachea tissues. Lung and trachea tissues were stained with H&E for evaluating infiltration of inflammatory cells followed thickness of epithelium. The OVA + PM group showed that thickness of epithelium was increased by infiltrating inflammatory cells compared with the normal saline (NOR) group. Treatment with dexamethasone reduced 23.17% and 58.9% in thickness of the pulmonary and tracheal epithelium of lung and trachea tissues, respectively. As shown in Figure 1A and B, epithelial thickness of the lung decreased by 31.32% in the BHT 629 group. Similarly, administration of 6.29 mg/kg, 62.9 mg/kg and 629 mg/kg of BHT attenuated epithelial thickness by 40.08%, 52.57% and 60.47% in trachea tissues (Figure 1A,C).

Lung and trachea tissues were stained with PAS for counting goblet cells, which secrete mucus in airway epithelial cells. Compared with the NOR group, production of PAS-positive goblet cells was markedly increased by 4.6 fold and *5*.5 fold in the OVA + PM group. Dexamethasone treatment decreased 73.17% and 39% of the number of goblet cells in lung and trachea tissues, respectively. In particular, goblet cell hyperplasia was significantly prevented, about 44.17% and 73%, by administration of BHT 629 mg/kg in the lung and trachea (Figure 1D,F,G). 

Lung tissues were stained with Masson′s trichrome for evaluating collagen deposition, expressing a vivid blue color in the peripheral bronchiole. In the OVA + PM group, the blue area of the bronchiole increased 6.7 fold in comparison with the NOR group. Treatment with dexamethasone lowered collagen accumulation by about 64.72%. Administration of 62.9 mg/kg and 629 mg/kg BHT alleviated fibrosis, reducing collagen for the blue area by about 41.53% and 80.17%, respectively, compared with the OVA + PM group (Figure 1E,H).

### 2.2. Effects of BHT on Inflammatory Cells in Bronchoalveolar Lavage Fluid (BALF) of OVA and PM_10_-Induced Mice

**Figure 2:**
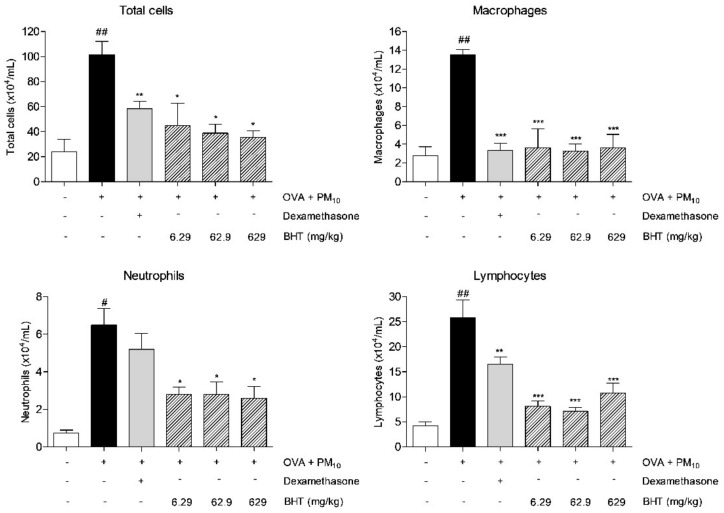
Total number of macrophages, neutrophils and lymphocytes in bronchoalveolar lavage fluid (BALF) of OVA + PM smaller than 10 μm (PM_10_)-induced mice. Each inflammatory cell was measured using a hemocytometer. Statistical results are presented as the mean ± SEM. ^#^ *p* < 0.05 and ^##^ *p* < 0.01 compared to NOR group; * *p* < 0.05, ** *p* < 0.01 and *** *p* < 0.001 compared to OVA + PM group.

We counted the number of white blood cells, including total inflammatory cells, macrophages, neutrophils and lymphocytes in BALF. In the OVA + PM group, releasing levels of total inflammatory cells and each white blood cell increased 4.3 fold, 4.9 fold, 8.7 fold and 6.1 fold compared with the NOR group. The administration of BHT (6.29 mg/kg, 62.9 mg/kg and 629 mg/kg) showed a concentration-dependent decrease by 55.83%, 61.74% and 65.12% in the number of total white blood cells of BALF. In particular, levels of macrophages, neutrophils and lymphocytes were significantly inhibited by about 73.33%, 60% and 58.25% by treatment of BHT 629 mg/kg (Figure 2).

### 2.3. Effects of BHT on Secretion of Serum Immunoglobulin Levels in OVA and PM_10_-Induced Mice

**Figure 3:**
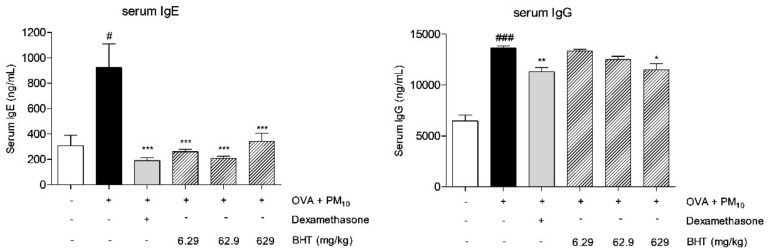
Total Immunoglobulin E (IgE) and Immunoglobulin G (IgG) levels in serum were measured in OVA + PM_10_-induced mice. Statistical results are presented as the mean ± SEM. ^#^ *p* < 0.05 and ^###^ *p* < 0.001 compared to the NOR group; * *p* < 0.05, ** *p* < 0.01 and *** *p* < 0.001 compared to OVA + PM group.

Expression levels of serum Immunoglobulin E (IgE) and Immunoglobulin G (IgG) were increased in the OVA + PM group 3 fold and 2.1 fold compared with the NOR group. When treated with dexamethasone, IgE and IgG levels decreased by 69.39% and 17.13% in comparison with the OVA + PM group. BHT administration (6.29 mg/kg, 62.9 mg/kg, 629 mg/kg) significantly suppressed generation of IgE by about 71.82%, 77.56% and 62.91%. BHT 629 mg/kg treatment reduced production of IgG by 15.51% (Figure 3).

### 2.4. Effects of BHT on JAK1/STAT6 Signal Pathway in OVA + PM_10_-Induced Mice and PM_10_-Treated A549

**Figure 4:**
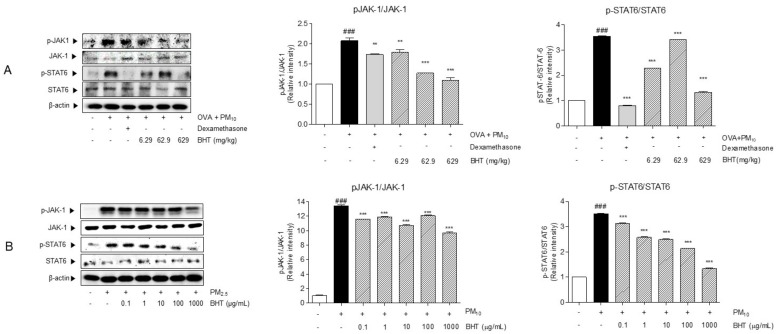
Protein levels of Janus kinase 1 (JAK1), phosphorylated JAK1, signal transducer and activator of transcription 6 (STAT6) and pSTAT6 were measured in (**A**) OVA + PM_10_-induced mice and (**B**) PM_10-_treated A549 cells. Quantitative data was analyzed by phospho-form/total-form ratio of JAK1 and STAT6. Statistical results are presented as the mean ± SEM. ^###^ *p* < 0.001 compared to the NOR group; ** *p* < 0.01 and *** *p* < 0.001 compared to OVA + PM group.

We measured protein levels of JAK1 and STAT6 in OVA + PM_10_-induced mice model by western blot. The protein expression tendency of phosphorylated JAK1 and STAT6 in the OVA + PM group significantly increased, by 2.08 folds and 3.5 folds, compared with the NOR group. BHT treatment suppressed levels of phosphorylated JAK1 about 13.89%, 38.83%, 47.19% compared with the OVA + PM treatment. Administration of BHT 629 mg/kg inhibited expression of the factor that promotes production of inflammatory cytokine, phosphorylated STAT6 by 62.57%, compared to the OVA + PM group (Figure 4A). 

In addition, we evaluated protein levels of JAK1 and STAT6 in PM_10_-treated A549 cells by western blot. The relative protein expression of phosphorylated JAK1 and STAT6 of PM_10_-treated A549 cells significantly increased, by 13.4 folds and 3.5 folds, in those of non-treated A549 cells in Figure 4B. BHT 1000 μg/mL treatment suppressed level of JAK1 about 27.96% compared with PM_10_ treatment. As presented in Figure 4, treatment of BHT dose-dependently inhibited expression level of phosphorylation of STAT6 by 10.95%, 26.59%, 29.18%, 39.28% and 61.92% in A549 cells (Figure 4B). 

### 2.5. Effects of BHT on Inflammatory Cytokines in OVA + PM_10_-Induced Mice and PM_10_-Treated A549

**Figure 5:**
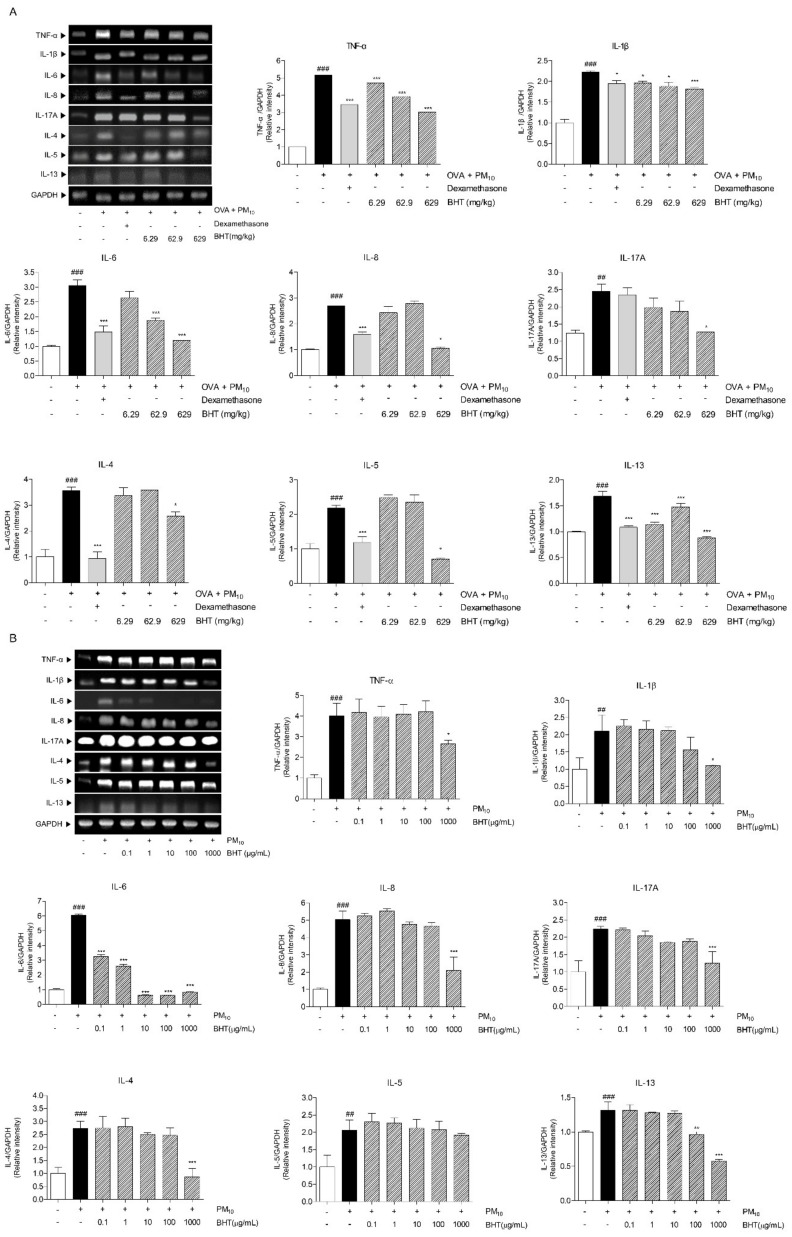
mRNA expression levels of inflammatory cytokines including tumor necrosis factor-α (TNF-α), interleukin (IL)-1β, IL-6, IL-8, IL-17A, IL-4, IL-5 and IL-13 were analyzed in (**A**) OVA + PM_10_-induced mice and (**B**) A549 cells. Statistical results are presented as the mean ± SEM. ^##^ *p* < 0.01 and ^###^ *p* < 0.001 compared to the NOR group; * *p* < 0.05, ** *p* < 0.01 and *** *p* < 0.001 compared to the OVA + PM group.

To determine inhibitory effects of BHT in airway inflammation, we investigated expression levels of pro-inflammatory cytokines, TNF-α, IL-1β, IL-6, IL-8 and IL-17A, which were analyzed by RT-PCR in OVA + PM_10_-induced mice. The levels of each pro-inflammatory cytokine were up-regulated about 5.2 folds, 2.2 folds, 3.1 folds, 2.7 folds and 1.7 folds in the OVA + PM group compared with the NOR group. Dexamethasone treatment significantly reduced levels of TNF-α, IL-1β, IL-6 and IL-8. Following treatment of BHT 629 mg/kg showed a significant decrease in levels of TNF-α, IL-1β, IL-6, IL-8 and IL-17A by 41.57%, 18.24%, 60.64%, 60.8% and 81.34% in dose-dependent manner. Th2-related cytokines, including IL-4, IL-5 and IL-13, were analyzed by RT-PCR in the same manner. The expression levels of each Th2-related cytokine were up-regulated by 3.6 folds, 2.2 folds and 1.7 folds in the OVA + PM group compared with the NOR group. Treatment with dexamethasone significantly inhibited production of Th2-related cytokines by 73.15%, 45.73% and 35.47%. Compared with the OVA + PM group, administration of BHT 629 suppressed levels of IL-4, IL-5 and IL-13 by about 27.57%, 67.57% and 47.75% (Figure 5A). 

To evaluate anti-inflammatory effects of BHT, the expression levels of pro-inflammatory cytokine were examined by RT-PCR in PM_10_-treated A549 cells. Levels of pro-inflammatory cytokine, TNF-α, IL-1β, IL-6, IL-8 and IL-17A were elevated in PM_10_-treated A549 cells by 4 folds, 1.3 folds, 6 folds, 5 folds and 2.2 folds, compared with non-treatment. BHT 1000 μg/mL treatment showed a significant decrease by 33.83%, 36.86%, 58.06% and 44.07% in expression of TNF-α, IL-1β, IL-8 and IL-17A. The expression levels of IL-6 were decreased by 46.16%, 57.18%, 89.57%, 89.98% and 86.09% in BHT 0.1, 1, 10, 100, 1000 μg/mL-treated group compared with PM_10_-treated A549 cells. Similarly, we investigated effects of BHT on Th2-related cytokines; the mRNA levels of IL-4, IL-5 and IL-13 were analyzed by RT-PCR. The Expression levels of Th2-related cytokine is increased by 2.7 folds, 2.1 folds and 1.3 folds in PM_10_-treated A549 cells compared with non-treated group. In comparison with PM_10_-treated A549 cells, expression levels of IL-4 and IL-13 is decreased 68.23%, 56.19% in BHT 1000 μg/mL treated A549 cells, respectively. However, no significant differences of IL-5 expression level were observed in BHT-treated A549 cells (Figure 5B). In summary, BHT treatment reduced level of several cytokines to untreated level or similar. These findings suggested that BHT reduced levels of pro-inflammatory and Th2-related cytokines, leading to regulation of inflammatory responses.

### 2.6. Effects of BHT on NF-κB Signal Pathway in OVA + PM_10_-Induced Mice

**Figure 6:**
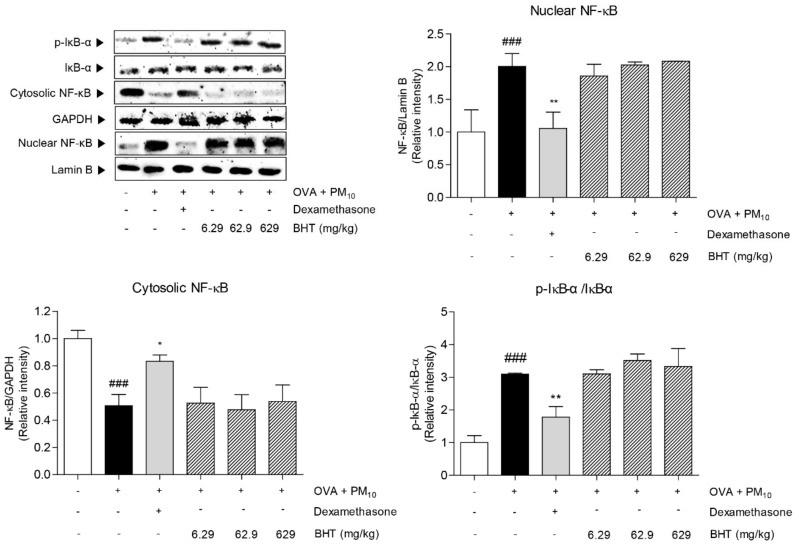
Protein levels of nuclear factor-kappa B (NF-κB) and phosphorylated inhibitor of nuclear factor-kappa B alpha (IκB-α) were measured in OVA and PM_10_-induced mice. Statistical results are presented as the mean ± SEM. ^###^ *p* < 0.001 compared to the NOR group; * *p* < 0.05 and ** *p* < 0.01 compared to the OVA + PM group.

We determined the effects of BHT on nuclear factor-kappa B (NF-κB) signal pathway in OVA + PM-induced mice by measuring level of NF-κB in nucleus and cytosol and inhibitor of nuclear factor-kappa B alpha (IκB-α) in cytosol. As shown in Figure 6, the expression level of NF-κB in nucleus was increased by 100.4% in the OVA + PM group compared with the NOR group. By contrast, the protein level of NF-κB in cytosol decreased by 49.35%. As the chain of NF-κB activation, expression protein level of phosphorylated IκB-α was elevated in the OVA + PM group by 3 folds compared with the NOR group. Treatment with dexamethasone significantly reduced expression levels of NF-κB activity. However, BHT treatment did not reach significance compared to OVA + PM group. 

## 3. Discussion

Allergic asthma is a complex and multifactorial disease, mainly characterized by airway obstruction, eosinophilic infiltration and airway remodeling. Recognition of allergens leads to immune responses, accompanying respiratory disturbance and bronchial hypersensitivity in airway [18]. Airway remodeling is accompanied with altered airway structure, hypersecretion of goblet cells and overgrowth of fibrillary collagen in allergic asthma [19]. Airway epithelium with disrupted structural integrity is permeable to allergen [20]. Thickness of airway epithelium is augmented in lung and trachea tissues, which is evidenced for the recruitment of inflammatory cells into bronchiole [21]. In addition, goblet cell hyperplasia is an underlying part of airway remodeling in asthma, which is featured by increasing the number of goblet cells and amount of mucus [22]. Mucus hypersecretion is observed in the airways under asthmatic conditions, according to PAS staining results [23]. Moreover, collagen deposition that is known as a typical histologic marker of asthma is connected with development of airway remodeling. Accumulation of collagen in the airways is related to promote dysfunction of lung in respiratory diseases. In this study, we demonstrated that BHT significantly reduced epithelial thickness and the number of goblet cells leading to suppression of producing mucus in airway tissues. Moreover, we confirmed that BHT has anti-fibrotic activity through blocking deposition of collagen in surrounding bronchiole lumen of OVA and PM_10_-induced mice model. 

Airway inflammation in allergic asthma is associated with the infiltration of major inflammatory cells, including macrophages, neutrophils and lymphocytes, into the bronchiole [24]. The activated inflammatory cells degrade components of the extracellular matrix and altered structure of airway [25,26]. In addition, hyperproduction of serum IgE and IgG is reported to exert allergic immune responses associated with T cells [27]. Th2-elicited cytokines, IL-4 and IL-13 is contributed to switch B-cell to inflammatory mediator, IgE, which is bound with mast cells that released histamine. IgG, which is a T-cell elicited antibody, is correlated with IgE levels [28]. Therefore, production level of IgE and IgG are important therapeutic marker to treat allergic asthma [28,29]. There was an increase on influx of inflammatory cells into the airway by allergen exposure, which is verified by elevation of total inflammatory cell counts in the OVA + PM group. BHT administration inhibited recruitment of inflammatory cells such as macrophages, neutrophils and lymphocytes into the bronchiole in allergic asthma mice. Moreover, total serum IgE and IgG levels were significantly reduced in BHT-treated groups compared to the OVA + PM group. 

Cytokine-mediated signal transduction in allergic asthma is triggered by JAK-STAT cascade [30]. The signal pathway induced by JAK1/STAT6 stimulated Th2 differentiation, which plays a critical role in regulating inflammatory gene expression in airway epithelial cells. Phosphorylated JAK1 activates the phosphorylation of STAT6, which translocate into the nucleus and regulate Th2-mediated inflammatory responses [31]. In particular, STAT6 is a pivotal transcription factor for generation of Th2-related cytokines, IL-4, IL-5 and IL-13 [32]. Recent research suggested that Th2 cell is considerably related with pathogenesis of allergic asthma [33]. Th2-derived cytokine, IL-4 was shown to be crucial to development for secretion of IgE in airway hyper-responsiveness condition. Another previous study illustrated that IL-5 is upregulated in OVA-induced mice models with elevating eosinophilic infiltration [33]. Moreover, IL-13 plays a key role in airway inflammation and is conceivable for asthma patients—this was evidenced with demonstration of an anti-IL-13 clinical test [34]. Furthermore, mechanisms behind allergic asthma were advanced since allergen-sensitized airway epithelial cells led to the recruitment of pro-inflammatory cytokines. Pro-inflammatory cytokines, such as of TNF-α, IL-1β, IL-6, IL-8 and IL-17A are concerned with the stimulation of airway epithelial cells to provoke airway wall destruction. Moreover, macrophages perceive allergens in the airway, thereby release pro-inflammatory cytokines to activate T-lymphocytes and expand Th2 cells [35]. TNF-α is secreted in macrophages, T-cells and epithelial cells that amplify asthmatic response. Expression of IL-1β and IL-6 are involved in production of IL-17A in inflammatory airway disease [36]. Other studies demonstrated that IL-8 is expressed in intensive asthmatic inflammation [37]. This study showed that BHT is an ameliorative remedy for inhibiting production of Th2-derived cytokines, such as IL-4, IL-5 and IL-13 by down-regulating JAK1/STAT6 signaling. Further, BHT down-regulated expression levels of pro-inflammatory cytokines, such as TNF-α, IL-1β, IL-6, IL-8 and IL-17A indicating that BHT has an effect on declining pro-inflammatory response for allergic asthma. Inhibition of pro-inflammatory and Th2-specific cytokines by BHT is consistent in the results from serum IgE and IgG reduction in BHT-treated mice.

Several signaling pathways, including NF-κB, JAK/STAT, oxidative stress and apoptosis are directly or indirectly associated with airway inflammation induced by PM. Predominant Th2-specific cytokines in allergic asthma are released by activation of NF-κB and JAK/STAT signaling. Previous studies have reported that NF-κB signaling is a potential factor for treatment in inflammation disease [38,39]. However, data from our results showed that BHT could not inhibit activation of NF-κB and degradation of Iκ-B-α. Taken together, BHT may not be involved in transcriptional expression of NF-κB activity, but regulates inflammation by targeting of JAK1/STAT6 signal transduction. 

BHT has been mainly prescribed for bronchial asthma as a traditional medicine as well as a health insurance medicine. Because six herbs are included in BHT, we guessed that a lot of active components may contribute to effects of BHT on airway inflammation. In particular, *Pinellia ternata* and *Magnolia officinalis* are major constitutive herbs in BHT, which have protective effects against allergic asthma [40,41]. Previous studies have reported that honokiol and magnolol isolated from *Magnolia officinalis* are able to alleviate inflammatory responses in allergic asthma [42,43]. Moreover, magnolol exerts anti-asthmatic effects by regulating the JAK/STAT pathway [44]. These references suggest that honokiol and magnolol, which are standard compounds of BHT, might be responsible constituents to improve allergic asthma. In addition, rosmarinic acid from *Perilla frutescens* effectively attenuates development of airway inflammation [45]. Further, 6-gingerol and 6-shogaol, major constituents of *Zingiber officinale,* significantly inhibit airway constriction and relax airway smooth muscle [46,47]. Furthermore, jujuboside B from *Ziziphus jujuba* possesses an anti-asthmatic activity on OVA-induced allergic asthma in mice [48]. 

In conclusion, BHT played an essential part in suppression of airway inflammation, mucus hypersecretion and airway remodeling through the JAK1/STAT6 signal pathway that modulates Th2-related immune response. BHT is expected to be a prospective herbal medicine for treatment of allergic asthma. Further study is needed to evaluate the beneficial effects of BHT as standardized medicine to prevent allergic asthma in humans.

## 4. Materials and Methods

### 4.1. Preparation of BHT

BHT (Lot. #702), a Korean Medicine for health insurance, was provided from Jungwoo Medicines Co., Ltd. (Seoul, Korea) BHT is composed of 382.5 mg of *Pinellia ternata*, 75 mg of *Magnolia officinalis*, 20 mg of *Poria cocos*, 100 mg of *Perilla frutescens*, 41 mg of *Zingiber officinale* and 244 mg of *Ziziphus jujube* dried powder extracts, based on the general requirements in the Korean Pharmacopoeia. Other additives, such as microcrystalline cellulose, calcium carboxymethylcellulose, silicon dioxide, magnesium stearate, hydroxypropylcellulose, polyethylene glycol 6000 and castor oil were included. The BHT tablet, 1020 mg, was finely ground and dissolved in distilled water.

### 4.2. Chemicals and Reagents

Sensitization and challenge for ovalbumin (OVA) (cat. #A5503) and aluminum hydroxide (AlOH_3_) (cat. #239186) were purchased from Sigma-Aldrich Corp. (St. Louis, MO, USA). In addition, urban dust (cat. #SRM 1649b), which is defined as particulate matters with smaller than 10 μm, was purchased from National Institute of Standards and Technology (Gaithersburg, MD, USA). Dexamethasone were purchased from Sigma-Aldrich Corp. 3-(4,5-Dimethylthiazol-2-yl)-2,5-Diphenyltetrazolium Bromide (MTT) (cat. #M6494) were purchased from Invitrogen; Thermo Fisher Scientific, Inc. (Bartlesville, OK, USA).

### 4.3. Animal Treatment

Five-week-old female BALB/c mice were purchased from Raonbio Inc. (Yongin, Korea). Mice were housed in a temperature and humidity controlled room on a 12 h light/dark cycle. The mice were acclimated to the environment for a week before experiments. All animal procedures were approved by the Committee on Care and Use of Laboratory Animals of the Kyung Hee University (KHUASP(SE)-19-098; Seoul, Korea). For in vivo experiments, mice were randomly divided into 6 groups (*n* = 7) as follows: (1) sensitization, challenged with normal saline (NOR), (2) sensitization with OVA, challenged with OVA and PM_10_ (OVA + PM), (3) sensitization with OVA, challenged with OVA and PM_10_ and orally administrated 200 μL of 3 mg/kg dexamethasone (DEX), (4) sensitization with OVA, challenged with OVA and PM_10_ and orally administrated 200 μL of 6.29 mg/kg BHT (BHT 6.29). (5) sensitization with OVA, challenged with OVA and PM_10_ and orally administrated 200 μL of 62.9 mg/kg BHT (BHT 62.9). (6) sensitization with OVA, challenged with OVA and PM_10_ and orally administrated 200 μL of 629 mg/kg BHT (BHT 629). The mice were sensitized with intraperitoneal injection of 100 mg OVA emulsified in 5 mg of AlOH_3_ as adjuvant in 1 mL saline on days 0, 7 and 14. On days 21, 22 and 23, the mice were challenged by intranasal instillation of 1 mg OVA combined with 100 μg PM_10_ in 50 μL normal saline. Dexamethasone and BHT (6.29, 62.9 and 629) in distilled water was administered orally once a day from start to end of experiment. There were no adverse effects or special note of BHT administration on mice in the experiment.

### 4.4. Bronchoalveolar Lavage Fluid (BALF) Analysis

On day 24, the mice were sacrificed after being anaesthetized with intraperitoneal injection of Rompun. Trachea was exposed by performing tracheostomy; a total of 2 mL bronchoalveolar lavage fluid (BALF) was withdrawn after injection of 3 mL PBS. BALF cell pellets were obtained through centrifugation at 17,000 rpm for 20 min. Supernatant were stored at −80 °C until use. Cell pellets were lysed with 20 μL of ammonium-chloride-potassium (ACK) lysing buffer (Sigma-Aldrich) twice, followed by centrifugation at 17,000 rpm for 10 min. After removing ACK buffer, the cell pellets were stained with 20 μL of giemsa solution. The number of total inflammatory cells, macrophages, neutrophils and lymphocytes was counted using a hemocytometer.

### 4.5. Serum Analysis

The day after the last challenge, mice were sacrificed and serum collected by cardiac puncture. Serum supernatant were obtained by centrifugation at 17,000 rpm for 20 min. The total serum IgE and IgG levels were measured by mouse enzyme-linked immunosorbent assay (ELISA) kit (cat. #555248, 552576; BD Biosciences, Franklin Lakes, NJ, USA). The 96-well plates were coated with 0.1 M Sodium Carbonate (pH 9.5) overnight. After blocking unspecific biding sites, diluted serum was loaded in each well and incubated for 2 h at room temperature (RT). Each well was washed for 5 times and added with detection antibody and streptavidin-horseradish peroxidase. 3,3’,5,5’-Tetramethylbenzidine substrate was added, thereby, the colored form was produced and optical density was detected by an ELISA reader at 450 nm.

### 4.6. Histopathological Analysis

After sacrifice, the lung and trachea were dissected and fixed in 10% formaldehyde. Dehydrated tissues were embedded in paraffin block and cut with 5 μm slices. Lung and trachea sections were stained with hematoxylin and eosin (H&E) and Periodic Acid-Schiff (PAS) kit (cat. #ab150680; Abcam plc., Cambridge, UK) to evaluate infiltration of inflammatory cells and measure goblet cell hyperplasia. Additionally, lung sections were assessed for collagen accumulation and lung fibrosis by Masson′s trichrome stain kit (cat. #25088; Polysciences, Inc., Warrington, PA, USA). The thickness of the epithelium, the number of goblet cells and proportions of collagen in each bronchiole were quantified by Image J program (ver. 1.38e; National Institutes of Health, Bethesda, MD, USA).

### 4.7. Cell Treatment

Human lung epithelial cell line, A549 cells, were purchased from Korean Cell Line Bank (Seoul, Korea) and culture in Dulbecco′s modified Eagle′s medium (DMEM) (Gibco; Thermo Fisher Scientific, Inc., Waltham, MA, USA) supplemented with 10% *v*/*v* fetal bovine serum (Gibco), 2 mM glutamine, 100 IU/mL penicillin and 100 μg/mL streptomycin (Gibco) at 37 °C in a 5% CO_2_ culture chamber. For in vitro experiments, A549 cells were treated with BHT at 0.1, 1, 10, 100, 1000 μg/mL blended with 100 μg/mL of PM_10_.

### 4.8. Cell Survival Assay

**Figure A1:**
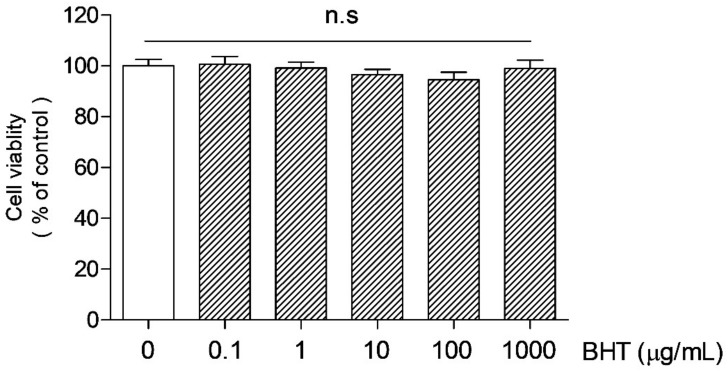
Cell viability of BHT treatment in A549 cells.

A549 cells were seeded at a density of 1 × 10^4^ cells/well in 96-well plates to 80% confluence. Concentration of BHT 0.1, 1, 10, 100 and 1000 μg/mL dissolved in PBS were treated for 24 h. After suction of medium, 50 μL of MTT solution was added to each well and incubated for 2 h. The medium was carefully removed, 50 μL of DMSO was dissolved for 30 min. Absorbance was measured by an ELISA reader at 570 nm. To calculate relative changes, background control was subtracted from all measurements. No cytotoxicity of BHT was observed in A549 cells (Figure A1).

### 4.9. Nuclear and Cytosolic Protein Fractionation

The nuclear and cytosolic fractions of lung tissues were extracted using lysis buffer. Lung tissues were incubated, homogenized with cytosolic buffer (10 mM HEPES, 10 mM KCl, 0.1 mM EDTA, 0.1 mM EGTA, 1 mM DTT, 0.15% NP-40, 50 mL β-glycerophosphate, 10 mL NaF, 5 mL Na₃VO₄, protease inhibitor) for 30 min and centrifuged at 17,000 rpm for 30 min. Cytosolic fractions were collected and lysed in nuclear buffer (20 mM HEPES, 400 mM NaCl, 1 mM EDTA, 1 mM EGTA, 1 mM DTT, 0.5% NP-40, 50 mL β-glycerophosphate, 10 mL NaF, 5 mL Na₃VO₄, protease inhibitor). The resulting nuclear and cytosolic fractions of NF-κB and IκB-α were analyzed by western blot.

### 4.10. Western Blot Analysis

Total protein of lung tissues and A549 cells were extracted using radioimmunoprecipitation assay (RIPA) buffer with protease inhibitor cocktails. Protein concentrations were quantified by the Bradford method. Moreover, 15 μg of protein were separated in 7.5% polyacrylamide gel, transferred onto polyvinylidene fluoride (PVDF) membranes. Membranes were blocked with 3% bovine serum albumin (BSA) at RT for 1 h and incubated with diluted primary antibodies (1:1000) at 4 °C for overnight. The primary antibodies were purchased from Santa Cruz Biotechnology (Santa Cruz, CA, USA) and Cell Signaling Technologies (Beverly, MA, USA). After removing primary antibodies, tris-buffered saline with Tween 20 (TBST)-washed PVDF membranes were incubated with horseradish peroxidase (HRP)-conjugated secondary antibodies from Santa Cruz Biotechnology at RT for 1 h. To quantify expression levels, membranes were reacted with enhanced chemiluminescence (ECL) reagents and detected by chemiluminescence imaging system (Young In, Seoul, Korea). The antibodies used were as follows: anti-β-actin (cat. #sc-47778), anti-glyceraldehyde-3-phosphate dehydrogenase (GAPDH) (cat. #sc-166574), anti-LaminB (cat. #sc-374015), anti-JAK1 (cat. #3332 S), anti-phosphorylated JAK1 (cat. #3331S), anti-STAT6 (cat. #sc-374021), anti-pSTAT6 (cat. #sc-136019), anti-NF-κB (cat. #3034S), anti-IκB-α (cat. #sc-1643), anti-pIκB-α (cat. #sc-8404), HRP-conjugated mouse anti-rabbit (cat. #sc-2357) and HRP-conjugated mouse-IgG_k_ (cat. #sc-516102).

### 4.11. Reverse Transcription-Polymerase Chain Reaction (RT-PCR)

Total RNA was isolated from lung tissue and A549 cells through TRIzol reagent. Homogenates were separated into aqueous phase and organic phase by chloroform after centrifugation. Aqueous phase and its same amount, 2-propanol, were gently inverted and centrifuged at 17,000 rpm for 15 min. Supernatants were discarded and RNA pellets were dissolved in RNase free water. The cDNA was synthesized with 1 μg of total RNA using Maxime RT PreMix kit (iNtRON Biotechnology, Inc.) (Seongnam, Republic of Korea) and each cDNA was amplified in final volume of 20 μL involving 2 μL cDNA, 10 pmol of specific forward primers, reverse primers and RNase free water by using Maxime PCR premix kit (iNtRON Biotechnology, Inc.). PCR products were separated by 1.5% agarose gel electrophoresis and photographed using unified gel documentation system (DAIHAN, Daegu, Republic of Korea). The primer sequences used in RT-PCR are listed in Table 1. The mRNA expression levels were normalized to GAPDH.

### 4.12. High-Performance Liquid Chromatography (HPLC) Analysis of BHT

**Figure A2:**
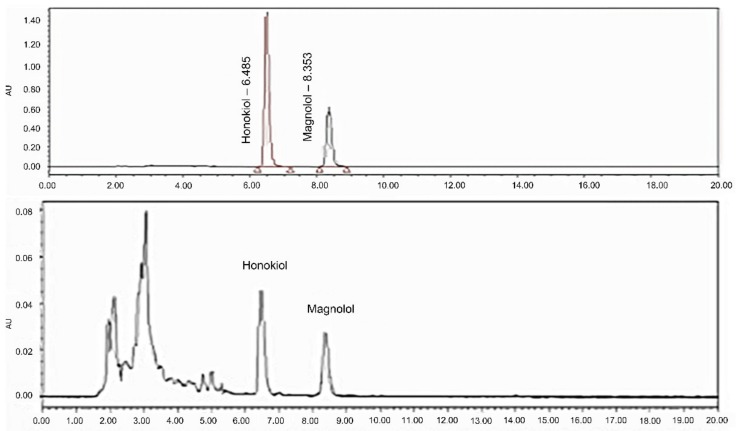
HPLC chromatograms of honokiol and magnolol in *Banhahubak*-tang tablet (BHT).

One gram of pulverized BHT tablet was extracted and filtered to make test liquid for injecting into the Alliance HPLC e2695 system with the 2489 UV/Visible detector. The standards of honokiol and magnolol were accurately weighed and dissolved in methanol. The peak of standard and sample solution were measured according to liquid chromatography as follows: separation of these analytes were done on a Capcell Pak C18 (I.D. 4.6 mm × L 25 cm). The mobile phases consisted of (A) CH_3_CN and (B) acetic acid (70:30). The mobile phase flow rate was 1.0 mL/min and the sample injection volume was 10 μL. Contents of honokiol and magnolol were 0.37 ± 0.01% and 0.20 ± 0.01%, respectively (*n* = 3) (Figure A2).

### 4.13. Statistical Analysis

All results were indicated as the means ± standard error of the mean (SEM). For comparison with data, one-way ANOVA followed by Tukey′s multiple test were used in these groups. The *p*-values were given as follows: * *p* < 0.05, ** *p* < 0.01, *** *p* < 0.001 were considered statistically significant.

## Figures and Tables

**Table 1:** Primer sequences used in RT-PCR.

Gene	Forward Primer (5′→3′)	Reverse Primer (3′→5′)
TNF-α	TACTGAACTTCGGGGTGATTGGTCC	CAGCCTTGTCCCTTGAAGAGAACC
IL-1β	CAGGATGAGGACATGAGCACC	CTCTGCACACTCAAACTCCAC
IL-6	CGGAGAGGAGACTTCACAGAGGA	GGAGAGCATTGGAAATTGGGG
IL-8	TGTGGGAGGCTGTGTTTGTA	TGTGGGAGGCTGTGTTTGTA
IL-17A	TCCAGAAGGCCCTCAGACTA	AGCATCTTCTCGACCCTGAA
IL-4	ATGGGTCTCAACCCCCAGC	GCTCTTTACGCTTTCCAGGAAGTC
IL-5	ATGATCGTGCCTCTGTGCCTGGAGC	CTGTTTTTCCTGGAGTAAACTGGGG
IL-13	ACCACGGTCATTGCTCTCA	GTGTCT CGGACATGCAAGCT
